# Electrically Accelerated Mechanochemical Film Formation by a Phosphonium Phosphate Ionic Liquid: An *In Situ* Chemical Kinetics Investigation

**DOI:** 10.1021/acs.langmuir.6c00931

**Published:** 2026-06-05

**Authors:** Foyez Ahmad, Ali Kaan Kalkan, Sharad Puri, David McIlroy, Huimin Luo, Jun Qu, Pranjal Nautiyal

**Affiliations:** † School of Mechanical and Aerospace Engineering, 7618 Oklahoma State University, Stillwater, Oklahoma 74078, United States; ‡ Department of Physics, 7618 Oklahoma State University, Stillwater, Oklahoma 74078, United States; § Manufacturing Science Division, 6146 Oak Ridge National Laboratory, Oak Ridge, Tennessee 37830, United States; ∥ Materials Science and Technology Division, 6146 Oak Ridge National Laboratory, Oak Ridge, Tennessee 37830, United States

## Abstract

Powertrains in electric vehicles are exposed to stray currents that accelerate wear and cause failure of mechanical components. These durability issues are further aggravated when using low-viscosity lubricants, which are desired for energy efficiency but create harsher contact conditions at sliding interfaces. This study investigates a phosphonium phosphate ionic liquid as a performance-enhancing additive in a low-viscosity base oil for lubricating electrified sliding interfaces. Ionic liquids can adsorb and react on contact interfaces via stress-assisted chemical reactions, generating nanometric *tribofilms* that provide protection against wear. However, the effect of electric fields on the mechanochemistry of ionic liquids is poorly understood, hindering their adoption in lubricants for electrified powertrains. This article reports an *in situ* optical interferometry study of ionic liquid derived tribofilm growth kinetics at stressed sliding/rolling interfaces under direct currents. The application of electric currents accelerated tribofilm formation up to a critical current density (∼1.4 A/mm^2^), beyond which pitting-induced wear dominated. The tribofilms were composed of iron phosphates, iron oxides, and carbon species, with iron oxides becoming predominant under applied currents. These tribofilms prevented the scuffing failure of steel surfaces under electrified conditions. Based on the results, a kinetic model is proposed that integrates electric current effect into the classical stress-assisted thermal activation framework to allow prediction of tribofilm growth at electrified sliding contacts. This framework provides crucial guidance for designing next-generation lubricants for electrified transportation and power generation systems.

## Graphical abstract

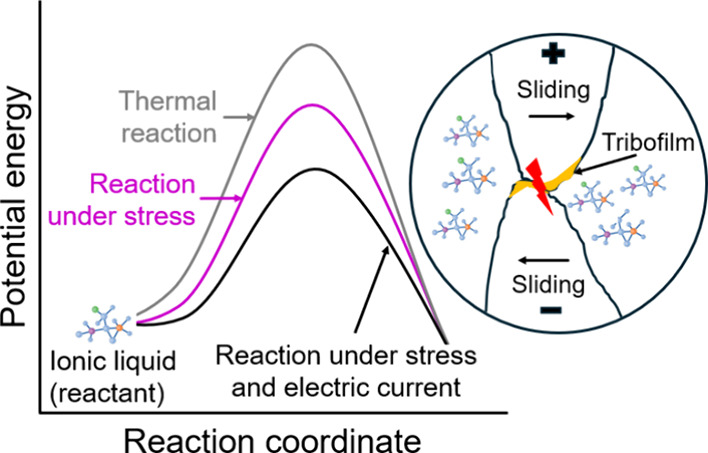

## Introduction

Drivetrains in electric vehicles (EVs) face unique lubrication challenges, since mechanical components like gears, bearings, and axles experience high torques, elevated temperatures, and unwanted stray currents. Stray currents, in concert with mechanical stresses, can initiate electro-tribological failure modes, such as electrical pitting, fluting, frosting, white etching crack, along with traditional adhesive and abrasive wear.^1,2^ Therefore, there is a critical need for advanced lubrication strategies to improve the durability of electrified drivetrains. Additionally, low-viscosity lubricants are essential for improving energy efficiency and driving range of EVs as they reduce churning losses.^3^ However, low-viscosity lubricants form thinner fluid films at sliding interfaces, increasing the likelihood of asperity contacts, which leads to elevated friction and wear.

To address these challenges, lubricants are formulated with performance additives^4^ to reduce friction and wear in boundary lubrication where surface asperities come into direct contact. These additives can react mechanochemically at stressed interfaces to generate nanometric tribofilms, which reduce friction and wear by preventing direct metal–metal contact.^5,6^ Ionic liquids, room-temperature molten salts composed of cations and anions, have emerged as promising lubricant additives owing to their strong surface adsorption, high thermal stability, and low flammability.^7^ The superior wear resistance of ionic liquids,^8^ coupled with their broad structural diversity,^9^ makes them well-suited for extreme conditions.^10^ A key advantage of ionic liquid is their multifunctionality, as they can simultaneously act as friction modifier, antiwear, and extreme pressure additives.^11^

As lubricant additives, ionic liquids function via two mechanisms, surface adsorption^12^ and tribofilm formation,^7^ depending on the contact pressure, temperature, molecular structure, and surface composition. At low contact pressures, the lubricity arises from ionic adsorption, where the polar heads of cations and anions attach to the metal surface, and the nonpolar alkyl tails align outward, forming ordered layers that reduce shear.^13,14^ On the other hand, under elevated temperatures and pressures, ionic liquids can decompose, and the reactive anions chemically react with the metal surface at sliding contacts.^15^ These mechanochemical reactions form protective tribofilms on metal surfaces, which enhances wear resistance. Among the diverse range of ionic liquids, the phosphonium phosphate ionic liquids possess excellent surface wettability, noncorrosiveness, high thermal stability, and the ability to form mechanically stable and thick tribofilms (from tens to hundreds of nm) that protect against wear.^11,16^ Therefore, this study focuses on a phosphonium phosphate ionic liquid.

Electrical conductivity is a critical property for designing lubricants for EVs. Conductive lubricants, such as ionic liquids, allow uniform current flow and help prevent arcing, while lubricants with low conductivity (below ∼ 4 × 10^–12^ S/cm) can cause static charge build-up and undesirable electric discharge at contacting surfaces.^17^ A notable feature of ionic liquids is their electrotunable interfacial behavior. Since ionic liquids are composed of mobile cations and anions, applied electric potential can modify the composition, arrangement, and dynamics of ions near the surface, thereby altering the interfacial structure and tuning friction.^18^ In recent years, ionic liquids have been studied as lubricants and lubricant additives for electrified sliding interfaces.^19–23^ Under modest electric fields, friction and wear tend to decrease as the applied potential promotes the adsorption of ionic species on the metal surface, forming stable interfacial films.^24,25^ However, higher currents cause the ion-adsorbed boundary layers to break down, which increases friction and surface wear.^24^ Electric fields also affect the formation of tribofilms through mechanochemical reactions.^22^ Lee et al.^23^ studied a protic ionic liquid, 2-hydroxyethylammonium 2-ethylhexanoate, in ultralow viscosity PAO2 oil and observed a strong dependence of tribofilm formation on applied current. Under moderate currents (0.1–0.5 A), the ionic liquid promoted the formation of carbonaceous tribofilms that significantly reduced wear by up to 84% compared to neat base oil without ionic liquid additive. Tuero et al.^26^ examined a phosphonium phosphate ionic liquid, trihexyltetradecylphosphonium bis­(2-ethylhexyl) phosphate, as an additive in automatic transmission fluid under direct currents of 0.5–1 A. Ionic liquid containing lubricant exhibited slightly improved surface protection due to the formation of tribofilms that suppressed abrasive wear. Overall, these studies highlight that the lubrication mechanisms under electrified conditions are highly dependent on the ionic liquid chemistry and the strength of the applied electrical currents. However, the mechanisms by which ionic liquids react and generate tribofilms under electric fields as well as the kinetics of these mechanochemical reactions remain poorly understood. These reactions occur at buried interfaces that are inaccessible to real-time observation, which makes it challenging to quantitatively resolve the underlying mechanochemical mechanisms.

To address these significant knowledge gaps, this article presents an in situ mechanochemistry study of ionic liquids at electrified contacts. We investigate the mechanochemical reactions of tetraoctylphosphonium bis­(2-ethylhexyl)­phosphate ([P_8888_]­[DEHP]) with steel surfaces at sliding/rolling interfaces under systematically varied direct electric currents. Using in situ optical interferometry, we quantified the growth kinetics of tribofilms as a function of the applied current. These measurements are used to develop a kinetic model that integrates electrical current into the classical stress-assisted chemical kinetics theory^27^ to predict tribofilm growth under the combined influence of electric fields and mechanical stresses. Additionally, ex-situ microscopic and spectroscopic techniques are employed to characterize the nanoscale structure and chemical composition of tribofilms to elucidate the chemical mechanisms driving tribofilm growth at electrified interfaces.

## Experimental Section

### Materials

Tetraoctylphosphonium bis­(2-ethylhexyl) phosphate ([P_8888_]­[DEHP]), with its molecular structure shown in Supporting Information Figure S1, was synthesized at Oak Ridge National Laboratory. Polyalphaolefin (PAO2) obtained from ExxonMobil was used as the base oil. One wt % of [P_8888_]­[DEHP] was blended in PAO2 by 5 min of magnetic stirring, followed by 30 min of bath sonication at room temperature. The ionic liquid-PAO2 mixture appeared visually homogeneous and remained stable without any signs of phase separation, which is consistent with previous studies showing phosphonium phosphate is soluble in PAO.^16^

### In Situ Mechanochemistry Experiments

Tribofilm growth experiments were conducted using the mini-traction machine tribometer, which allows independent control of ball and disc rotation. In this test, a 52100 steel ball (19.05 mm diameter) was loaded against a flat 52100 steel disc, both with RMS surface roughness of ∼ 3–5 nm. The normal load was set at 75 N, which corresponds to a maximum Hertzian contact pressure of around 1.29 GPa. Details of the Hertzian contact pressure calculations are provided in the Supporting Information (Section S2.1). To study tribofilm growth, a 2-h constant-speed test was performed at a mean rolling speed of 240 mm/s and 10% slide-roll ratio (SRR), which defines the extent of sliding and rolling at the contact (see Supporting Information Section S2.5). To investigate current-induced effects on tribofilm formation, a programmable DC power supply was used to apply a controlled electric field across the contact. At least three tests were performed for each test condition. Tribofilm growth kinetics was measured *in situ* using the Spacer Layer Imaging Method (SLIM), described in detail in the Supporting Information (Section S2.6).

### Tribofilm Characterization

The FEI Quanta 600 field emission scanning electron microscopy (SEM), equipped with Bruker QUANTAX EDS detector, was used to examine both the surface morphology and elemental composition of the selected tribofilms at an acceleration voltage of 20 kV.

An atomic force microscope (AFM, MFP3D Infinity, Santa Barbara, CA, USA) operating in contact mode was used to obtain high-resolution topographic images of tribofilms and quantify surface roughness. A pyramidal silicon tip (Model: HQ:CSC17/Al BS, MikroMasch) with a resonant frequency of 13 kHz and a force constant of 0.18 N/m, was employed to acquire AFM images.

Chemical characterization of the selected tribofilms was conducted using an X-ray Photoelectron Spectroscopy (XPS) system (XR40B-EC X-ray source, Prevac; EAC2000–125 hemispherical analyzer, Omicron) operated in an ultrahigh vacuum chamber at ∼ 3 × 10^–10^ Torr. The Mg Kα emission line from a dual-anode source was used at 345 W power, and photoemitted electrons were detected with the hemispherical analyzer. A 1 mm diameter spot was focused during data acquisition, and surface compositions were determined from the peak areas of Fe 2p, O 1s, C 1s, and P 2p core-level spectra. An iterated Shirley background was applied for background subtraction, and a symmetric Gauss–Lorentz product line shape was used for curve fitting.

Raman spectroscopy was performed using a WITec alpha 300 R Raman microscope, employing 532 nm laser excitation, a 100-μm confocal aperture (fiber) diameter, a 600 lines/mm grating, and a 100× objective lens of 0.90 numerical aperture. The laser power and beam spot size on the samples were set to 4.9 mW and 1 μm, respectively. The signal was integrated for 30 s. At these conditions, the Raman spectra were reasonably stable and free of photothermal shifts in peak positions. We present the Raman spectra after baseline subtraction (asymmetric least-squares smoothing method using Origin 2024).

## Results and Discussion

### Tribofilm Growth Kinetics

Tribofilm growth tests were carried out using a ball-on-disc tribometer which generates mixed sliding/rolling contacts, shown in Figure 1a. An AISI 52100 steel ball was loaded against an AISI 52100 steel disc, both submerged in the ionic liquid containing synthetic oil (PAO2), which was maintained at 100 °C throughout the test. The normal and tangential stresses at the ball/disc interface induced tribofilm formation. During the tests, direct current was applied across the ball/disc contact to investigate the effect of electric field on tribofilm growth. Tribofilm growth kinetics was measured *in situ* using the SLIM method,^28^ as illustrated in Figure 1b. The test was periodically paused to capture interference images of the tribofilm (Figure 1c), described in detail in the Supporting Information (see Section S2.6).

**Figure 1:**
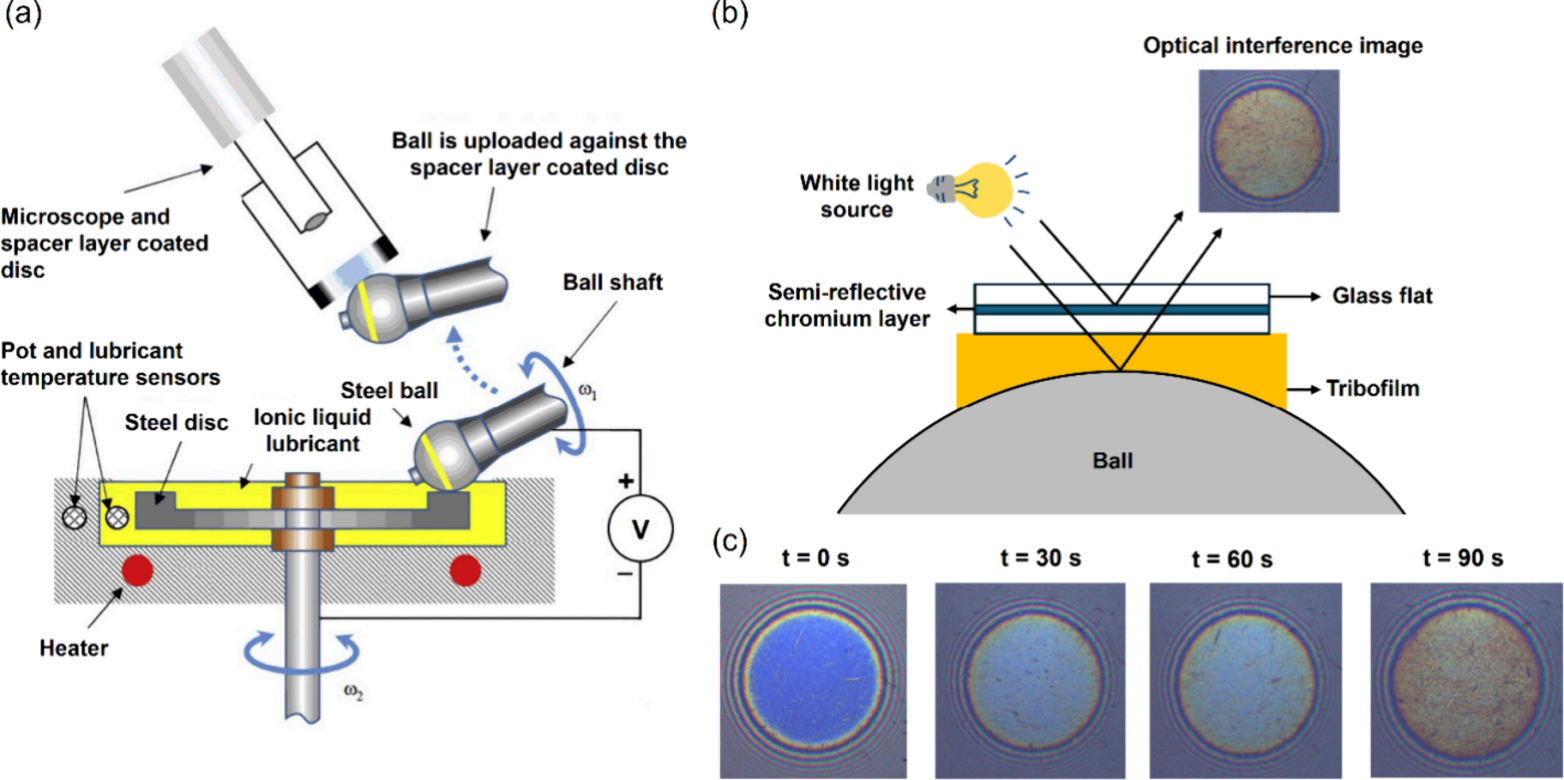
(a) Schematic of an *in situ* ball-on-disc tribometer used for studying mechanochemical film growth under controlled electrified conditions, (b) Working principle of the Spacer Layer Imaging Method (SLIM) used to optically measure tribofilm thickness evolution (adapted from ref 29. Available under a CC-BY 4.0 license. Copyright © 2019, Dawczyk, J., Russo, J. & Spikes, H.), and (c) A series of *in situ* SLIM images showing tribofilm nucleation and growth on a ball specimen.

The time-dependent evolution of tribofilm thickness under different electric currents (0–200 mA) is shown in Figure 2a. A ∼ 3–5 nm thin tribofilm was observed at 0 A. However, when direct current was applied, tribofilms thickness rapidly increased, reaching ∼ 10–25 nm within the first 2 min (the SLIM images are shown in Figure S3). This initial rapid growth can be attributed to the high reactivity of the phosphonium phosphate ionic liquid with the steel surface.^15^ Once the available reactive sites are covered by the tribofilm, continued contact cycles cause tribofilm removal. Ultimately, a final thickness of 5–10 nm was reached after ∼ 40 min, when the rates of tribofilm growth and removal processes became roughly equal. This dynamic between tribofilm growth and removal also influenced friction behavior. The traction coefficient increased initially as the tribofilm developed, then gradually declined as the film was removed, as shown in Figure S4.

**Figure 2:**
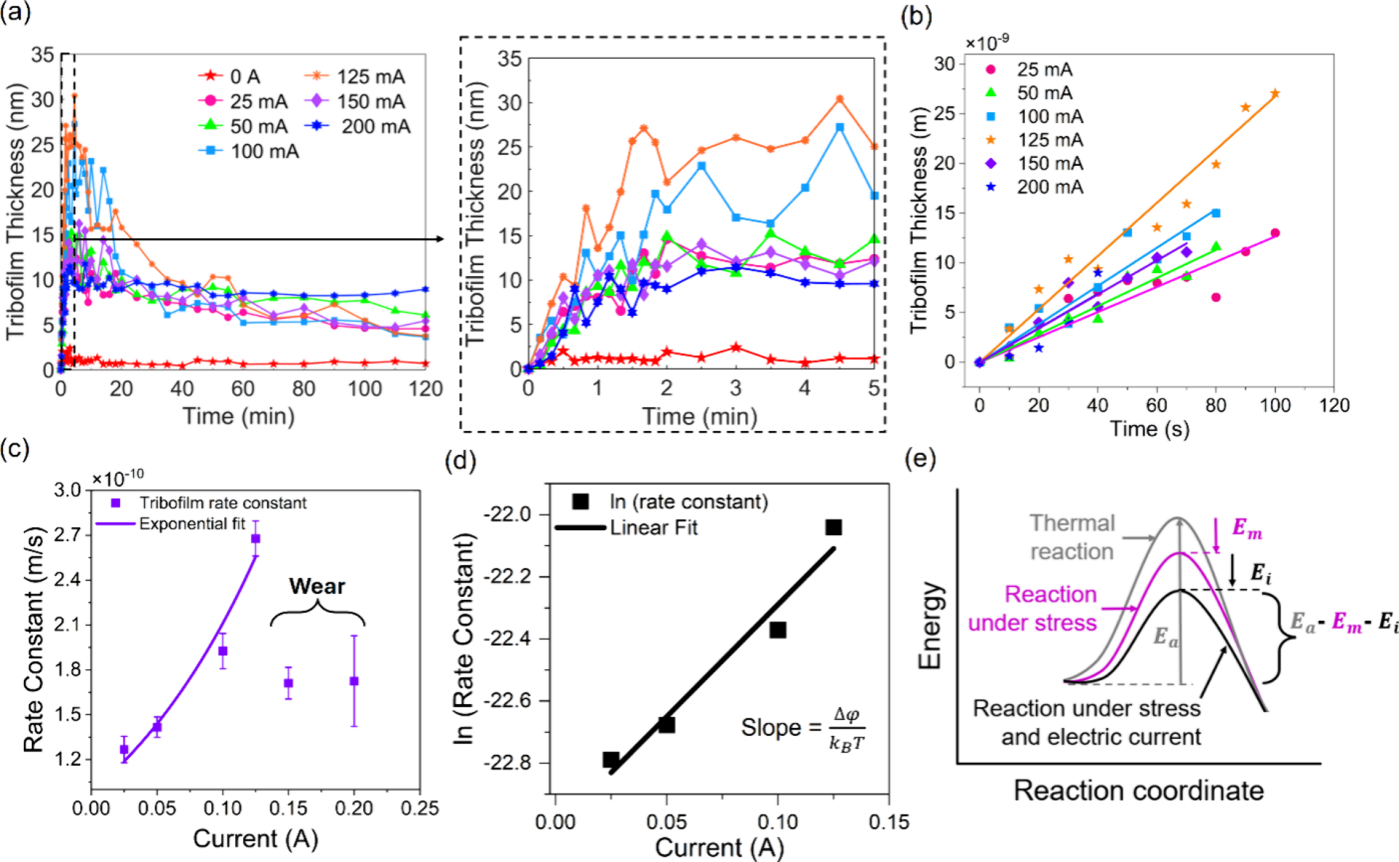
Tribofilm growth under varying electric currents: (a) Evolution of tribofilm thickness with time under applied currents ranging from 0 to 200 mA; zoomed inset magnifies the initial 5 min to show tribofilm growth behavior. (b) Linear fit applied to quantify the initial tribofilm growth rate. (c) Dependence of rate constant on applied current. (d) Semilog plot of reaction rate constant vs current was used to calculate the activation constant (Δφ) for electrically assisted tribofilm growth reaction. (e) Simplified schematic of the potential energy surface (PES) showing the effects of stress and electric current on the energy barrier for tribofilm growth reactions.

In contrast, tests conducted with neat PAO2 base oil (without the ionic liquid additive) resulted in scuffing (adhesive wear) under both electrified and unelectrified conditions, accompanied by a sharp spike in traction and pronounced surface damage (see Supporting Information Figures S4 and S5). These observations highlight the critical role of ionic liquid derived mechanochemical films in preventing failure at sliding interfaces.

In Figure 2b, linear fits were applied to the initial growth regime to calculate tribofilm growth rates. Since the tribofilm growth is linear with time, we assume zero-order kinetics where the rate of reaction (i.e., the rate of tribofilm growth) equals the rate constant, *k* (see Supporting Information Section S3.1 for details). As shown in Figure 2c, the extracted rate constants increased exponentially with applied direct current up to 125 mA. Beyond this point, a decline in growth rate was observed with further increase in current. This reduction may be attributed to competing electrically induced wear mechanisms^2^ which could undermine tribofilm formation. Previous work on zinc dialkyldithiophosphate (ZDDP) showed suppressed tribofilm formation under all electrified conditions, with severe localized anodic wear at higher applied voltage (5 V) whereas much less wear occurred at 1 V.^30^

Tribofilm growth at stressed interfaces is driven by the coupling of mechanical and thermal energy, which together help reactants to overcome the energy barrier required for mechanochemical reaction. The kinetics of tribofilm growth is described by the stress-assisted thermal activation model:^27^

$$k=A\cdot \exp\left(-\frac{E_{a}-E_{m}}{k_{b}T}\right)=A\exp\left(-\frac{E_{a}-\sigma \Delta V}{k_{B}T}\right)$$ 1

where *A* is the pre-exponential factor, *k*_*B*_ is the Boltzmann’s constant, *T* is the absolute temperature, *E*_*a*_ is the thermal activation energy, and *E*_*m*_ is the mechanical energy. The rate constant, *k* is exponentially related to the effective energy barrier (*E*_*a*_ – *E*_*m*_) that must be overcome for the reaction to proceed. For a stress-assisted reaction, *E*_*m*_ can be expressed as a product of contact stress (σ) and an activation constant, named activation volume (*ΔV*). *ΔV* quantifies how effectively applied stress lowers the activation energy barrier for the reaction.^31^

At electrified interfaces, the applied electric currents supply additional energy, which may further promote the reaction. This could lead to Joule heating, which will promote mechanochemical reactions through thermal activation. We calculated the temperature rise due to Joule heating (see Supporting Information Section S2.3), which is estimated to be merely ∼ 2 °C at 125 mA. This suggests there are additional non-Joulean effects, which may decrease the energy barrier for mechanochemical reactions.^32,33^ To illustrate this concept, the potential energy surface (PES) along a hypothetical reaction coordinate is schematically presented in Figure 2e. This can happen if the current injects electrons into molecules into antibonding orbitals, making it easy to break the bonds. Electron-induced bond dissociation in hydrocarbon molecules has previously been shown using scanning tunneling microscopy.^34^ The electric field may also ionize molecules in the lubricant due to dielectric breakdown,^35^ making them more reactive. Alternatively, the applied electrical energy could increase the potential energy of the reactant(s) by stretching bonds.^36^ This increase in potential energy of the reactants will also reduce the effective energy barrier for the reaction.

Based on this conceptual framework, the effect of electric current on reaction kinetics can be expressed by the following Arrhenius-type equation,

$$k=A\exp\left(-\frac{E_{a}-E_{m}-E_{i}}{k_{B}T}\right)=A\exp\left(-\frac{E_{a}-\sigma \Delta V-I\Delta \varphi }{k_{B}T}\right)$$ 2

where *E*_*i*_ represents the contribution of electrical current in reducing the energy barrier. Taking analogy from the mechanical energy term in eq 1, we express *E*_*i*_ as a product of the applied current, *I*, and a new activation parameter, *Δφ*, which describes the sensitivity of the activation energy barrier to electric current.

Taking the natural logarithm of eq 2 yields the following expression,

$$\ln\left(k\right)=\ln A-\frac{E_{a}}{k_{B}T}+\frac{\sigma \Delta V}{k_{B}T}+\frac{I\Delta \varphi }{k_{B}T}$$ 3

Based on this expression, we can derive *Δφ* from the slope of ln­(*k*) vs *I* plot for the early stage tribofilm growth regime, as shown in Figure 2d. Using the experimental data reported in Figure 2, *Δφ* for the tribofilm growth reactions was calculated to be 3.7 × 10^–20^ J/A. By quantifying *Δφ*, we establish a direct correlation between applied current and reaction kinetics. We propose this activation parameter could be useful to compare the electrically induced reactivity of lubricant additives and obtain insights into the molecular mechanisms of tribofilm formation at electrified interfaces. A similar kinetic model has also been proposed for voltage-assisted tribopolymerization of ambient hydrocarbons at nanoscale sliding contacts by Qu and Carpick, where film growth was governed by applied voltage rather than current.^36^ Our future studies will investigate the relationships between *Δφ* and the molecular structure of ionic liquids.

### Morphology and Chemical Composition of Tribofilms

To understand the effect of the electric currents on tribofilm morphology, we conducted scanning electron microscopy (SEM) of tribofilms formed on disc specimens under 0, 100, and 150 mA test conditions. In the absence of electric current, scratches along the sliding direction were seen (Figure 3a), which represent the typical morphology of abrasive wear. Upon the application of electric current, the running track appears smoother and more uniform, as further supported by AFM-based surface roughness measurements provided in the Supporting Information (Figure S6). This suggests rapid tribofilm formation due to electric currents protected the surfaces from abrasive wear. However, microsized pits were observed on tribofilms formed under electrified conditions (Figure 3b,c). The formation of these micron-sized pits may be related to electrical discharges, which cause intense local heating and melting of the surface material.^37^ When the molten material comes into contact with the surrounding lubricant, it undergoes rapid quenching, resulting in hardened, brittle edges around the melt pool. These brittle edges are highly susceptible to fracture and spallation under continued sliding or rolling conditions.^38^ The current densities in our experiments under 100 and 150 mA conditions are calculated to be ∼ 1.14 and 1.71 A/mm^2^, respectively (see Supporting Information Section S2.2), which are higher than the literature reported value of 0.53 A/mm^2^ required to induce surface damage.^37^ This suggests that the applied currents in our study are likely sufficient to cause similar discharges and contribute to the observed pitting. In addition to these electric discharge-driven effects, sliding in electrochemically active environments can also promote pitting due to tribocorrosion, which may further intensify under electrified conditions.^39,40^ The pitting density was quantified by calculating the ratio of the pitted area to the total measured area (Figure 3d). Although pitting was observed under all test conditions (see Figure S7), it dominated at higher current densities, increasing exponentially with applied direct currents (Figure 3d). This observation suggests tribofilm growth and pitting mechanisms compete at electrified interfaces. Therefore, we conclude that the suppression of tribofilm growth observed under currents exceeding 125 mA (shown in Figure 2c) is due to pitting.

**Figure 3:**
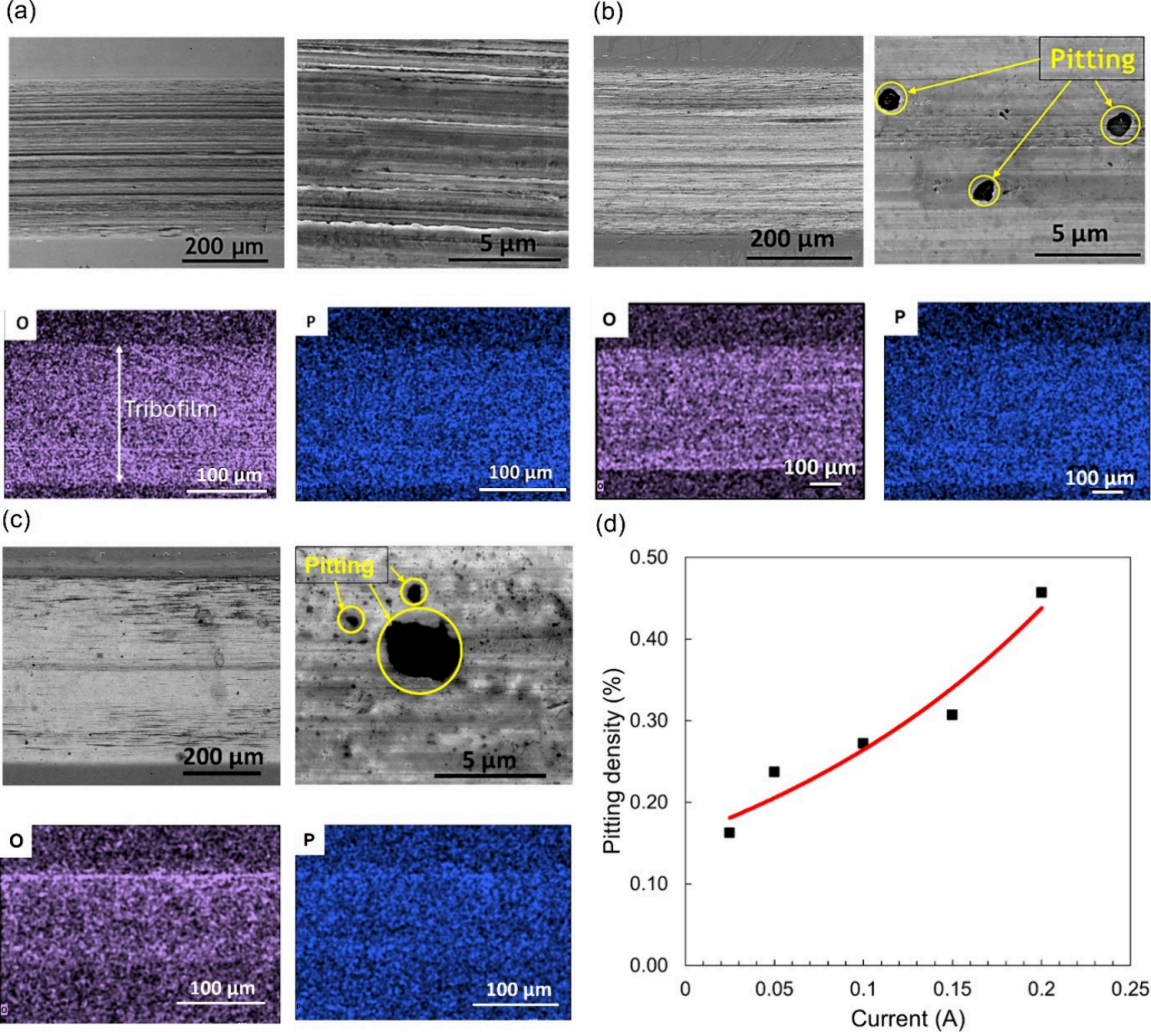
Morphology and composition of tribofilms generated at (a) 0 mA (unelectrified), (b) 100 mA, (c) 150 mA test conditions. For each condition, SEM micrographs of the tribofilm at two different magnifications are shown in the top row. The EDS elemental maps for oxygen (O) and phosphorus (P) are shown in the bottom row. (d) Pitting density as a function of applied current, showing exponential dependence on applied current.

Energy-Dispersive X-ray Spectroscopy (EDS) analysis revealed the presence of phosphorus and oxygen elements in the tribofilms under both electrified and nonelectrified conditions. A higher concentration of P and O elements was detected only in the Hertzian contact area (running track), which was subjected to contact stresses and electric currents. These results confirm the formation of a phosphonium phosphate derived tribofilm due to mechanical stresses and electrical currents.

To gain deeper insight into the chemical mechanisms of tribofilm formation, XPS was employed due to its superior surface sensitivity. Figure 4 presents the XPS core-level spectra of Fe 2p, C 1s, O 1s, and P 2p collected from the tribofilms formed on the disc surface under unelectrified and electrified (150 mA) test conditions. The Fe 2p spectra for both tribofilms consist of the 2p_3/2_ and 2p_1/2_ peaks at 710.5–711.2 eV and 723.7–724 eV, respectively, which correspond to Fe^2+^ or Fe^3+^ oxidation states or a mixture thereof.^41^ No signal attributable to metallic Fe^0^ is observed. This result indicates that the surface iron exists in oxidized form, confirming the presence of oxides in the tribofilm. The C 1s spectrum for the unelectrified tribofilm shows a main peak at 285.4 eV from C–C bonds^9^ and a smaller peak at 288.3 eV from COOH groups.^42^ In contrast, the C 1s spectrum for the tribofilm formed at 150 mA shows a distinct peak at 286.5 eV associated with C–O bonding,^42^ which suggests the formation of oxidized carbon species during tribofilm formation. The additional peak at 290.3 eV could not be assigned definitively. The O 1s spectrum for the unelectrified tribofilm shows a broad peak at 531.5 eV corresponding to O–C bonding,^43^ along with smaller peaks at 529.9 and 533.4 eV that can be attributed to O–Fe bonding^9^ from iron oxides and O=P bonding^44^ from phosphate compounds, respectively. In contrast, the O 1s spectrum from the electrified tribofilm includes two distinct features: A peak at 533.3 eV corresponds to O=P or C–O functional groups,^43,44^ while a second peak at 537.5 eV likely represents the contaminant and/or the unreacted ionic liquid. The P 2p spectra for both tribofilms presents a well-defined peak at 133.2–133.4 eV, which is consistent with P–O bond.^9^ This indicates the formation of phosphate compounds, most likely iron phosphates,^45,46^ resulting from mechanochemical reaction between phosphonium phosphate and the steel surface. Additional unidentified peak in the electrified tribofilm (∼143 eV) is attributed to possible contamination or unreacted ionic liquid, as this peak is consistent with one of the peaks observed in the XPS spectrum of neat ionic liquid on the silicon substrate provided in the Supporting Information (Figure S8). Altogether, the spectra suggest the tribofilms are composed of iron oxides, iron phosphates, and oxidized carbon species, formed via mechanochemical reactions under both electrified and nonelectrified conditions.

**Figure 4:**
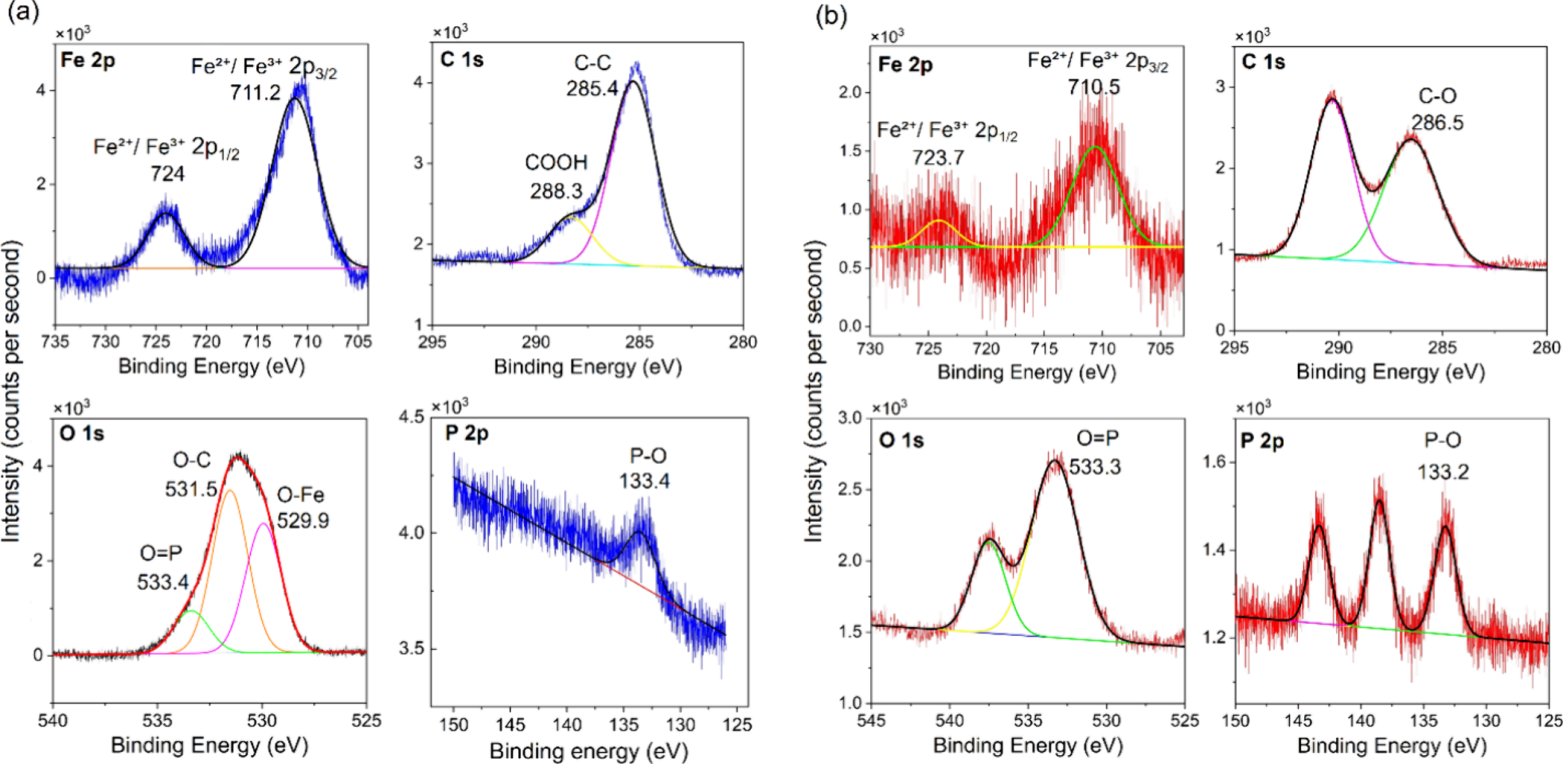
XPS core-level spectra of ionic liquid derived tribofilms on steel disc produced under (a) unelectrified and (b) electrified (150 mA) test conditions.

Raman spectroscopy was also performed to characterize the chemical structure of tribofilms formed under 0 and 150 mA electrified conditions (Figure 5). Magnetite (Fe_3_O_4_) peaks^47,48^ were observed at 481 and 664 cm^–1^ in both tribofilms, with an additional peak at 306 cm^–1^ in the unelectrified tribofilm. Additionally, peaks are detected at 291 and 1330 cm^–1^ for the tribofilm obtained under electrified conditions only. These peaks indicate the presence of hematite (Fe_2_O_3_)^48,49^ and suggest the formation of mixed iron oxides on the electrified rubbing surfaces. Peaks observed between 1007 and 1012 cm^–1^ are attributed to the stretching vibrations of PO_4_ tetrahedra,^50^ consistent with the presence of FePO_4_ (iron phosphate).^51–53^ These features suggest that phosphate species derived from the ionic liquid anion, chemically react with the steel surface to form phosphate-based tribofilm. An earlier study has also reported that ionic liquid decomposition products contribute to phosphate-based tribofilm formation on rubbing surfaces.^15^

**Figure 5:**
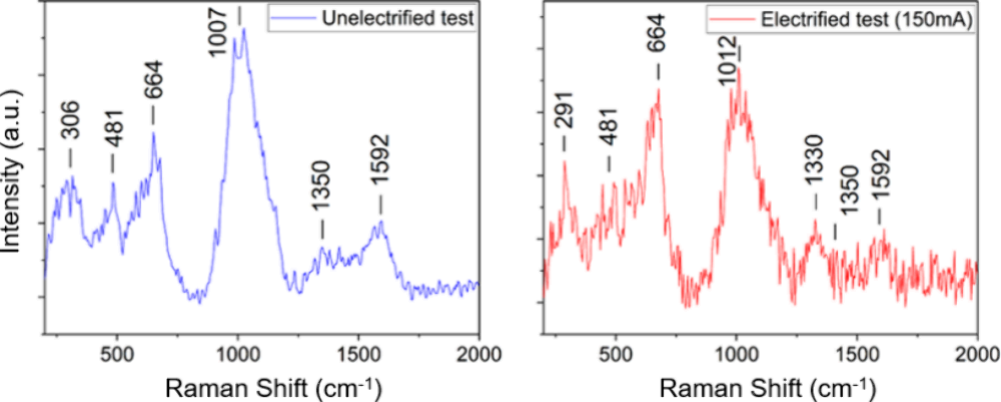
Raman spectra of ionic liquid derived tribofilms formed under unelectrified (left) and electrified (right) test conditions.

The increased relative intensity of magnetite peaks compared to phosphate peaks^53^ near 1000 cm^–1^ under electrified conditions may be attributed to enhanced oxidation processes driven by localized Joule heating and electrical discharges at asperity contacts. Alternatively, the oxidation may be electrochemical as a result of electron transfer from Fe to a reduction reaction. In the unelectrified case, the contact surface forms magnetite in which Fe exists in mixed +2/+3 states. On the other hand, under electrification, Fe is oxidized to the +3 state, forming hematite. Thus, the extent of oxidation is higher in the electrified condition. A similar voltage dependent oxidation behavior has been observed in ionic liquid mediated electrochemistry at AFM tip–substrate contacts, where increasing bias voltage promoted the formation of Fe^3+^ rich iron oxide films.^54^

The two other peaks observed at 1350 cm^–1^ (D-band) and 1592 cm^–1^ (G-band) are characteristic of sp^2^-carbon-related species^55^ formed from the decomposition of organic compounds in the ionic liquid. While these compounds may contribute to friction reduction, they may also act as hard, abrasive soot particles.^56^

Overall, these results suggest that iron oxide formation is the primary driver of the enhanced tribofilm growth at electrified interfaces. Controlled oxidation can lead to a protective iron oxide tribofilm on steel; however, excessive oxidation may promote wear due to the release of abrasive oxide particles from the surface.

### Mechanisms of Tribofilm Formation

The application of electric current influences tribofilm formation through complex mechanochemical reactions at electrified sliding/rolling contacts. In our study, the tribofilm growth rate increased exponentially with applied direct current up to 125 mA (Figure 2). These electrically accelerated mechanochemical reactions can be attributed to two synergistic effects: (1) enhanced adsorption of ionic species at the metal surface^57,58^ and (2) enhanced ion mobility under the electric field.^59^ A prior computational study on phosphonium phosphate ionic liquid showed that electric field induced molecular mobility promotes tribochemical reactions by directing reactive species toward the surface.^59^ Electric field could also enhance reactions by stabilizing the transition state, thus lowering the reaction barrier.^60^ Such effects have been observed in the electrostatic catalysis of Diels–Alder reactions.^32^ In addition, a molecular dynamics study on alkyl phosphate additives showed that external electric fields significantly increased molecular decomposition, which is the rate limiting step for mechanochemical tribofilm growth.^61^

Although different from ionic liquids, a recent nanoscale study on the tribopolymerization of ambient hydrocarbons provides further insight into the role of electric fields in accelerating mechanochemical reactions.^36^ In that work, tribopolymer growth increased exponentially with applied voltage, as electric fields lowered the activation barrier. The authors proposed an electric field induced bond-stretching mechanism in which the electric field acted on polar bonds, pulling them toward rupture and thereby facilitating faster mechanochemical reactions.^36^ This mechanism could also have contributed to the decomposition of ionic liquid molecules in our experiments, accelerating tribofilm growth. Notably, the tribopolymerization study reported that the reaction kinetics was independent of current, whereas tribofilm growth in our study is correlated with current. This suggests that there are additional mechanisms underlying current-assisted mechanochemical reaction in our study. For instance, injection of electrons into antibonding orbitals could promote bond dissociation, promoting tribofilm formation.

We discovered that the tribofilms formed under electrified conditions are oxide-rich, with a higher content of Fe^3+^ compared to the unelectrified conditions. This observation is consistent with a previous study on commercial gear oils and automatic transmission fluids which reported enhanced oxidation at electrified sliding interfaces.^56^ The formation of the protective iron oxide-containing tribofilm in our study is mediated by the phosphonium phosphate ionic liquid. In contrast, tests performed with just the base oil (without the ionic liquid) did not produce a protective tribofilm and instead resulted in severe scuffing of the steel surfaces (see Figure S5).

At higher current levels (>125 mA), tribofilm growth was suppressed and did not follow the exponential dependence as shown in Figure 2c. Three mechanisms may explain this deviation in our study: (1) excessive currents lead to localized electric discharges and initiate pitting due to Joule heating,^38,62^ (2) electrically accelerated oxidation of steel and subsequent release of hard oxide particles from the surface may lead to three-body abrasive wear mechanism,^63^ and (3) tribocorrosion may also promote surface pitting,^39^ which could undermine tribofilm growth. These observations highlight that tribofilm growth under electric fields is governed by competition between mechanochemical reactions and wear in the mixed lubrication regime. Future studies under full-film elastohydrodynamic lubrication conditions^31^ could better isolate tribofilm growth from wear and provide more controlled reaction conditions.

## Conclusions

We reported the influence of direct electric currents on mechanochemical film formation by a phosphonium phosphate ionic liquid used as an antiwear additive in a synthetic low viscosity oil. Using *in situ* optical interferometry, we quantified the growth kinetics of tribofilms at stressed and electrified sliding-rolling contacts with nanoscale resolution. Tribofilm growth rate increased exponentially with applied current up to a current density of ∼ 1.4 A/mm^2^, beyond which pitting-induced wear dominated and suppressed tribofilm formation. The tribofilms were primarily composed of iron phosphates, iron oxides and carbon species, with a higher proportion of iron oxides under electrified conditions, suggesting electrically accelerated tribofilm growth is driven by surface oxidation. This tribofilm protected steel surfaces from scuffing failure (adhesive wear) at electrified sliding-rolling contacts. Our results show that the formation of this oxide-rich tribofilm was mediated by the ionic liquid additive, as testing with just the neat oil (without ionic liquid) did not generate a protective tribofilm and exhibited scuffing. A kinetic model based on stress-assisted reaction rate theory was proposed to describe the current-dependent tribofilm growth behavior. A new activation parameter is introduced to quantify the susceptibility of the reaction barrier to applied currents, providing a predictive framework for designing mechanochemically active lubricants for electrified interfaces. Our future research will probe and link this activation parameter with the chemical structure of lubricant additives to reveal the molecular mechanisms underpinning the mechano-electrochemically driven tribofilm growth reactions. Such insights will inform the design of advanced lubricants for electrified automobiles, aircraft, and energy devices operating under extreme electrical and mechanical conditions.

## Supplementary Material



## Notes

Additional details are provided in the Supporting Information, including materials, methods, supplementary text, and Figures S1–S8 (PDF)
