# Supplementary material for: Electrically Accelerated Mechanochemical Film Formation by a Phosphonium Phosphate Ionic Liquid: An In Situ Chemical Kinetics Investigation
Source: Langmuir. 2026 Jun 5;42(23):16422–31. doi: 10.1021/acs.langmuir.6c00931 (PMC13276892; doi:10.1021/acs.langmuir.6c00931)
Supplement: Supplementary file 1 [file la6c00931_si_001.pdf]

Supporting Information

Electrically Accelerated Mechanochemical Film Formation by a Phosphonium Phosphate Ionic Liquid: An *In-situ* Chemical Kinetics Investigation

Foyez Ahmad[1], Ali Kaan Kalkan[1], Sharad Puri[2], David McIlroy[2], Huimin Luo[3], Jun Qu[4], Pranjal Nautiyal[1,*]

[1]School of Mechanical and Aerospace Engineering, Oklahoma State University, Stillwater, Oklahoma, USA, 74078

[2]Department of Physics, Oklahoma State University, Stillwater, Oklahoma, USA, 74078

[3]Manufacturing Science Division, Oak Ridge National Laboratory, Oak Ridge, Tennessee, USA, 37830

[4]Materials Science and Technology Division, Oak Ridge National Laboratory, Oak Ridge, Tennessee, USA, 37830

*Corresponding Author: pranjal.nautiyal@okstate.edu

Content:

S1. Materials

S2. Methods

S3. Supplementary Text

S4. References

S1. Materials

The key physical properties of the ionic liquid and the base oil used in this study are summarized in **Table S1**. The molecular structure of the ionic liquid is shown in **Figure S1**.

Table S1. Properties of polyalphaolefin base oil (obtained from the vendor) and tetraoctylphosphonium bis(2-ethylhexyl)phosphate ([P_{8888}][DEHP]) ionic liquid

Lubricant	Density (g/cm^3)	Viscosity at 100°C (cP)
Ionic liquid additive, [P_{8888}][DEHP]	0.86	68.2
Base oil, PAO2	0.75	1.3

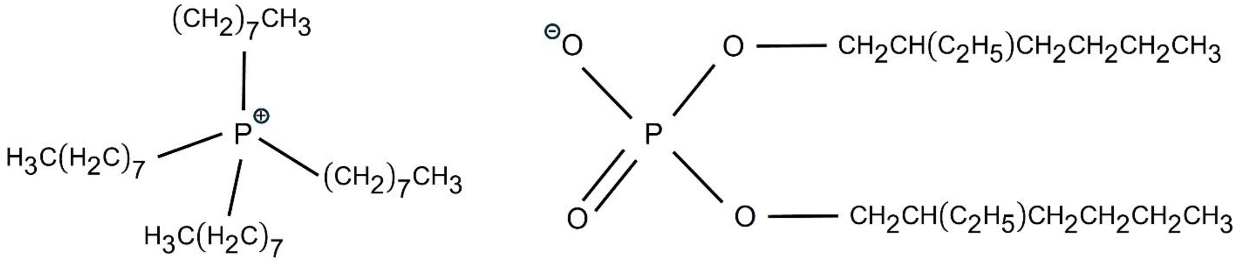

Figure S1. Molecular structure of [P_{8888}][DEHP] ionic liquid

S2. Methods

In-situ tribofilm growth experiments were conducted using a Mini Traction Machine (MTM, PCS Instruments, London, UK), which consists of independently rotating ball and disc specimens, and allows precise control of load, speed, slide-roll ratio, and lubricant temperature. The MTM tribometer is equipped with an optical interferometer, which permits in-situ measurement of tribofilm formation on ball specimens. The tribofilm growth tests in this work were conducted at 75 N load, 100℃ temperature, 240 mm/s mean/rolling speed, and 10% slide-roll ratio. Additionally, direct currents ranging from 25 to 200 mA were passed through the ball/disc contact, to probe the effect of electric fields on mechanochemical reactions. Additional information regarding the selection of test conditions is provided below.

S2.1. Calculation of the Hertzian contact pressure

The maximum Hertzian contact pressure, σ_{max} and the mean Hertzian contact pressure, σ_{mean}, for the given test conditions were calculated using the following equation,

$$\sigma_{\mathrm{max}} = \frac{3F}{2\pi a^2} \quad \text{(S1)}$$

$$\sigma_{\mathrm{mean}} = \frac{F}{\pi a^2} \quad \text{(S2)}$$

$$a = \left(\frac{3FR}{4E'}\right)^{1/3} \quad \text{(S3)}$$

where F is the normal load, R is the ball radius, a is the Hertzian contact radius and E' is the reduced Young's modulus. Reduced Young's modulus, E' is given by,

$$\frac{1}{E'} = \left(\frac{1-v_A^2}{E_A} + \frac{1-v_B^2}{E_B}\right) \quad \text{(S4)}$$

where v_A and v_B are Poisson's ratios, and E_A and E_B are the Young's moduli of contacting bodies A and B, respectively.

The test conditions used in this study yielded a maximum Hertzian contact pressure of 1.29 GPa.

S2.2. Calculation of electric current density at the contact

Current densities were calculated based on the applied direct current (I) and the Hertzian contact area (A),

$$J = \frac{I}{A} = \frac{I}{\pi a^2} \quad \text{(S5)}$$

The Hertzian contact radius (a) is 167 μm (derived using Eq. (S3) above). Accordingly, the current densities for the applied currents in this study are summarized in **Table S2**.

Note: A denotes the apparent contact area. The true contact area is smaller due to surface roughness.

Table S2. Current densities for the applied direct currents in this study

Electric current (mA)	Current Density (A/mm^2)
25	0.29
50	0.57
100	1.14
125	1.43
150	1.71
200	2.28

S2.3. Calculation of interfacial temperature rise due to frictional and Joule heating

The temperature rise at the electrified sliding/rolling contact was estimated by considering two sources of heat generation: frictional heating generated by sliding at the contact, and Joule heating generated by current flow across the contact. The total heat generation rate, $\dot{Q}$ is therefore written as,

$$\dot{Q} = \dot{Q}_f + \dot{Q}_j \tag{S6}$$

Frictional heating rate, $\dot{Q}_f$ is given by

$$\dot{Q}_f = \mu F U \tag{S7}$$

where μ is the measured traction coefficient (plotted in **Figure S4**), F is the applied normal load, and U is the rolling (mean) speed.

Joule heating rate, $\dot{Q}_J$ is given by

$$\dot{Q}_J = IV \tag{S8}$$

where I is applied current and V is the measured voltage drop across the ball and disc interface.

The mean flash temperature rise for a circular Hertzian contact was then estimated using the following equation,[1]

$$\theta_{mean} = \frac{\dot{Q}}{aK} \frac{0.3654}{\sqrt{J+1.299}} \tag{S9}$$

where a is the half diameter of the contact (which is Hertzian contact radius; see Eq. (S3)) and K is the thermal conductivity of steel. The dimensionless speed parameter is defined by, $J = \frac{U\,a}{\chi}$; here χ is the thermal diffusivity of the steel.

Using Eq. (S6)-(S9), the mean flash temperature rise at the 125 mA electrified interfaces is calculated as 72.6 °C (we used μ = 0.12; see **Figure S4**). However, only Joule heating induced mean temperature rise was estimated to be about 2.1 °C. Therefore, Joule heating is expected to make only a negligible contribution to the interfacial temperature rise under the present test conditions.

S2.4. Determination of lubrication regime

The degree of asperity contacts at sliding/rolling interfaces plays a crucial role in tribofilm growth reactions. Depending on the thickness of the lubricant film relative to the surface roughness, a system may operate in one of three lubrication regimes: boundary, mixed, or elastohydrodynamic. In the boundary regime, most of the load is supported by direct asperity contacts; in the elastohydrodynamic regime, a full fluid film separates the surfaces in motion; and in the mixed regime, both the fluid film and asperity contacts share the applied load. In this study, the lubrication regime was identified by conducting a Stribeck test[2]. In this test, the traction coefficient at a loaded and lubricated sliding contact was measured as a function of mean rolling speed (see **Figure S2**). Based on this measurement, a rolling speed of 240 mm/s (mixed lubrication region) was selected for tribofilm growth experiments shown in the main paper (**Figure 2)**. We selected the mixed regime in this study because the milder asperity interactions are ideal for studying contact-induced mechanochemical reactions, without inducing severe wear (which can dominate in the boundary regime).

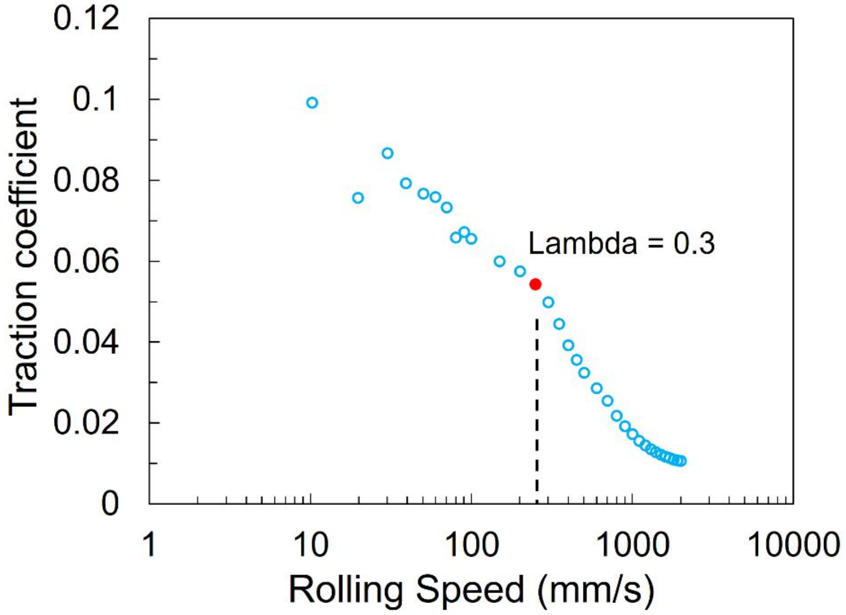

Figure S2. Stribeck curve showing traction coefficient as a function of mean rolling speed for a steel-steel contact lubricated with PAO2 oil additized with a phosphonium phosphate ionic liquid.

The extent of asperity interactions at sliding/rolling contacts can be quantified by a dimensionless parameter, λ, which represents the ratio of the lubricant film thickness to the composite root mean square (RMS) roughness of the two contacting surfaces:

$$\lambda = \frac{h_0}{\sqrt{\sigma_1^2+\sigma_2^2}} \tag{S10}$$

where h_0 is the minimum lubricant film thickness, and σ_1 and σ_2 are RMS surface roughness of the ball and disc surfaces. The minimum lubricant film thickness at the contact was calculated using the Hamrock–Dowson model for point contacts,[3]

$$\frac{h_0}{R'} = 3.63\left(\frac{U\eta_0}{E'R}\right)^{0.68}(\alpha E')^{0.49}\left(\frac{W}{E'R}\right)^{-0.073}\left(1 - e^{-0.68k}\right) \tag{S11}$$

In this formulation, U is the mean/ rolling speed (also referred to as entraining speed), η_0 is the dynamic viscosity of the lubricant, E' is the reduced modulus, α is the pressure–viscosity coefficient, W is the applied load, and k is the ellipticity parameter (k=1 for point contact).

Pressure-viscosity coefficient is calculated as,[4]

$$\eta_p = \eta_0 e^{\alpha p} \tag{S12}$$

where η_p refers to the dynamic viscosity of the lubricant at a given pressure (p), and η_0 is the dynamic viscosity of the lubricant at atmospheric pressure.

Using the above equations, the λ value was calculated to be 0.3, which corresponds to the mixed lubrication regime.[5]

S2.5. Slide-roll ratio

The slide-roll ratio (SRR), which defines the extent of sliding and rolling at the contact, is given by

$$\mathrm{SRR} = \frac{U_D - U_B}{U} \quad \text{(S13)}$$

where U_D and U_B are the speed of the disc and ball, respectively. The mean rolling speed, U is calculated as,

$$U = \frac{U_D + U_B}{2} \quad \text{(S14)}$$

The tribofilm growth tests in this study were performed at 10% SRR.

S2.6. In-situ optical interferometry

Tribofilm growth kinetics was measured *in situ* using the Spacer Layer Imaging Method (SLIM).[6] In this method, the test is periodically stopped, the steel ball is pressed against a glass window and illuminated with white light. The glass window is coated with a semi-reflective chromium layer that partially reflects the incident light. The remaining light passes through the spacer layer and tribofilm, reflects off the steel substrate, and interferes with the light reflected at the glass interface (illustrated in **Figure 1b**). The resulting interference pattern provides a spatially resolved map of tribofilm thickness on the ball surface (as shown in **Figure S3)**. A refractive index of 1.6 was assumed to convert optical path differences into tribofilm thickness, consistent with values reported in previous studies on phosphate-based tribofilms.[7,8] SLIM measurements were performed following the standard MTM SLIM calibration procedures.[6] Before each test, a zero image was collected using a dry stationary ball-on-glass contact to determine the spacer layer thickness and set the optical conditions. The white light intensity was adjusted during this step so that the zero image thickness remained in a physically reasonable spacer layer thickness range (120±10 nm). The light intensity and camera settings were then kept unchanged throughout the tests. In the image analysis, a consistent sampling region was chosen since the reported thickness can be sensitive to the analysis area.

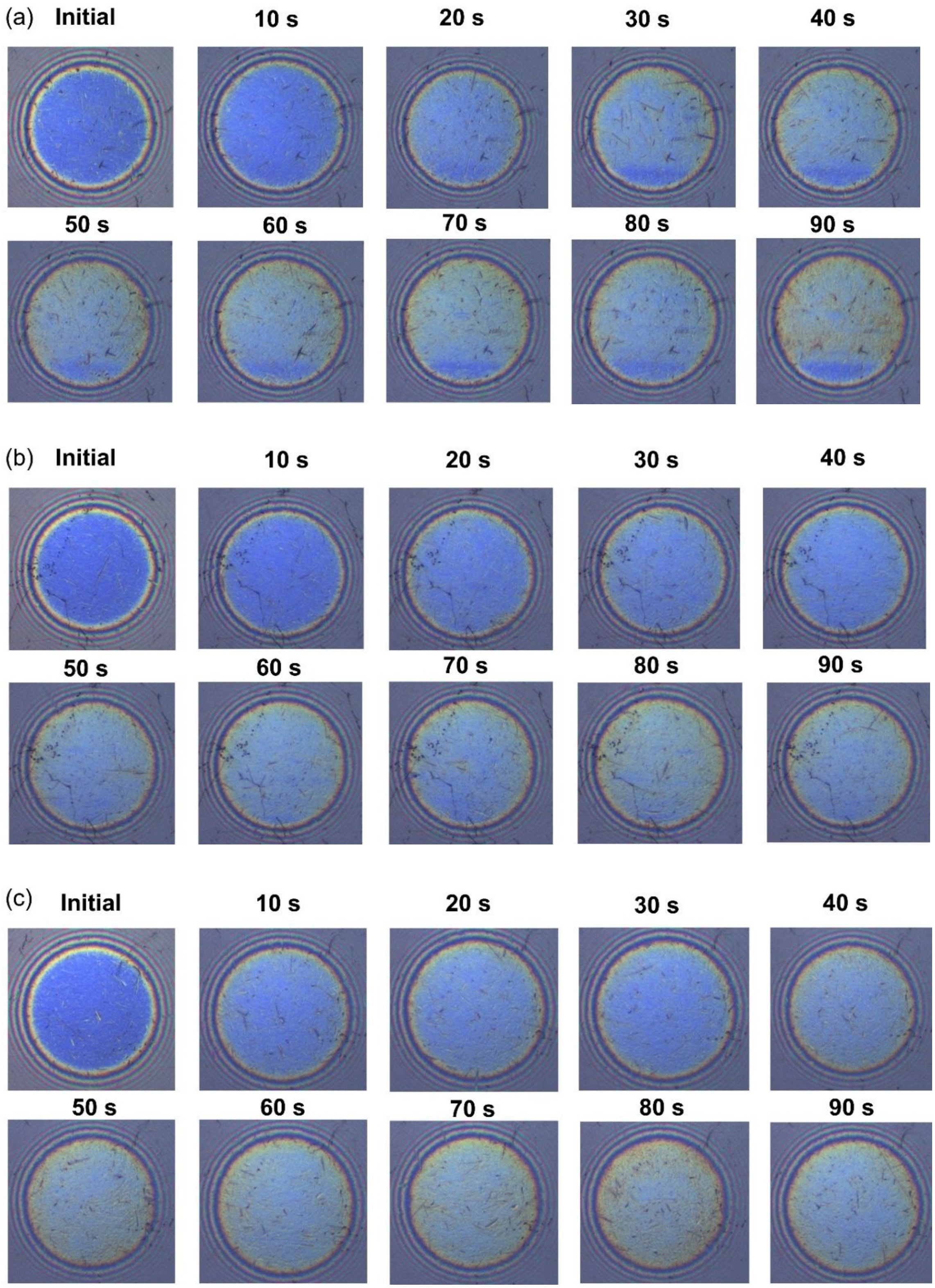
(a) Initial
10 s
20 s
30 s
40 s
50 s
60 s
70 s
80 s
90 s
(b) Initial
10 s
20 s
30 s
40 s
50 s
60 s
70 s
80 s
90 s
(c) Initial
10 s
20 s
30 s
40 s
50 s
60 s
70 s
80 s
90 s

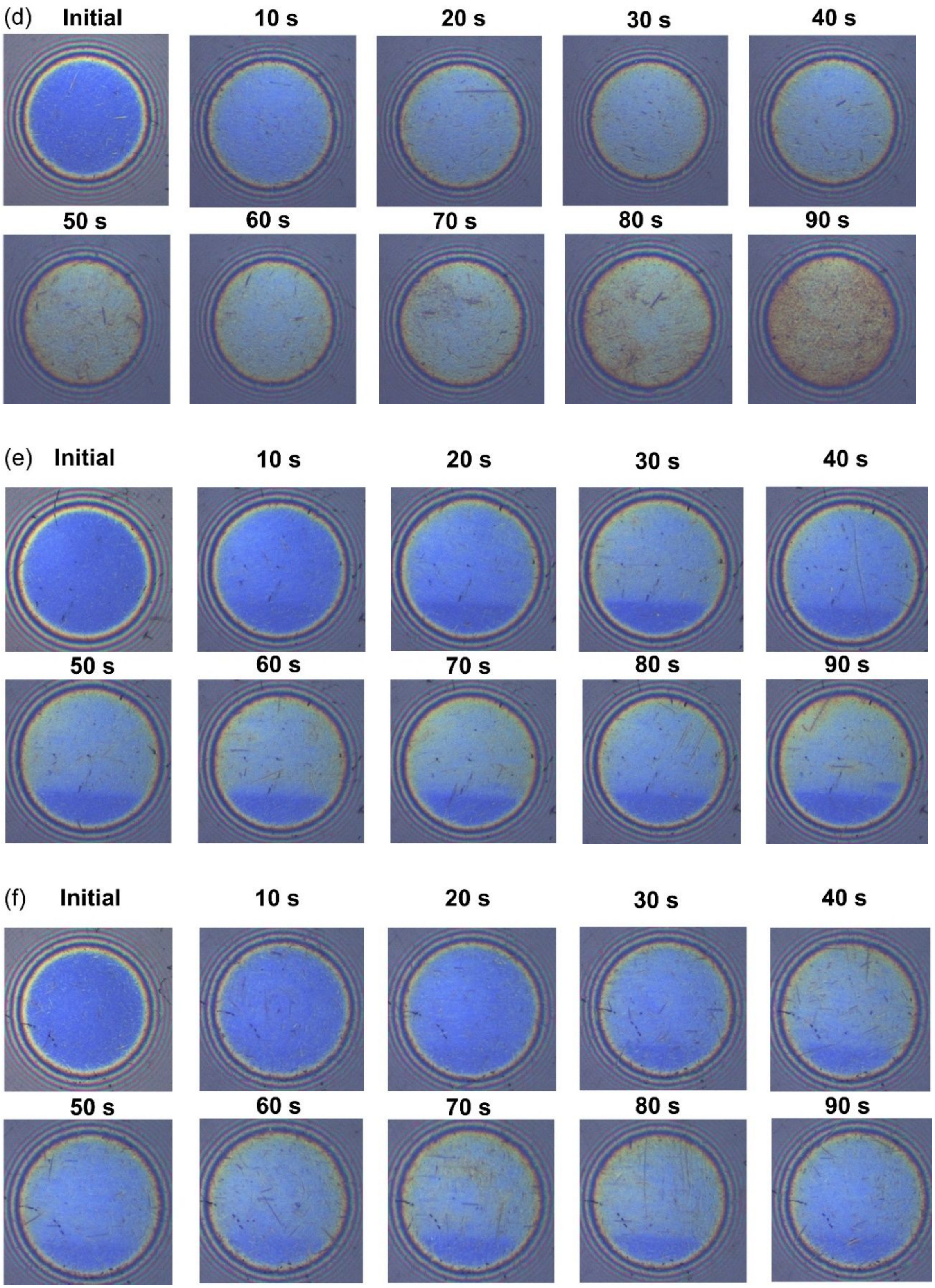

Figure S3. Series of optical interference images showing progressive tribofilm growth on the ball surface under (a) 25 mA, (b) 50 mA, (c) 100 mA, (d) 125 mA, (e) 150 mA and (f) 200 mA test conditions.

S3. Supplementary Text

S3.1. Reaction order

The rate of tribofilm growth can be expressed as an nth order reaction:

$$\frac{d[h]}{dt} = k[h]^n \qquad \text{(S15)}$$

where n is the reaction order, $[h]$is the tribofilm thickness, k is the rate constant, and t is time.

For a zero-order reaction, the integrated rate law becomes,

$$[h] = [h]_0 + kt \qquad \text{(S16)}$$

As shown in **Figure 2b**, the initial tribofilm growth is linear with respect to time, akin to Eq. (S16). Therefore, we assume tribofilm growth reactions in our study follow zero-order kinetics. The slope of this linear region represents the rate constant, k. The rate constants reported in **Figure 2c** were obtained by linear fitting of the initial tribofilm growth regime. Similar kinetic analysis for tribofilm growth reactions has previously been reported for ZDDP derived tribofilms by Gosvami et al.[9]

S3.2. Antiwear functionality of phosphonium phosphate tribofilm

Phosphonium phosphate ionic liquids exhibited superior antiwear protection compared to neat PAO2. **Figure S4** shows traction vs. time plots for neat PAO2 as well as PAO additized with ionic liquid. Tests with PAO2 exhibited scuffing under both electrified and unelectrified conditions (**Figure S4)**. Scuffing refers to a sudden failure of lubricated sliding contacts characterized by a sharp rise in friction, temperature, and surface damage due to localized welding or plastic flow at asperity contacts.[10,11] In tests with neat PAO2, a sudden spike in traction was observed, followed by rapid specimen seizure due to severe scuffing. Scuffing was exacerbated under the 200 mA test condition, where failure occurred within 2 minutes. In contrast, ionic liquid-based formulations exhibited a stable and declining traction coefficient over test durations across all current levels. This behavior highlights the superior antiwear performance provided by ionic liquid derived tribofilms under electrified conditions.

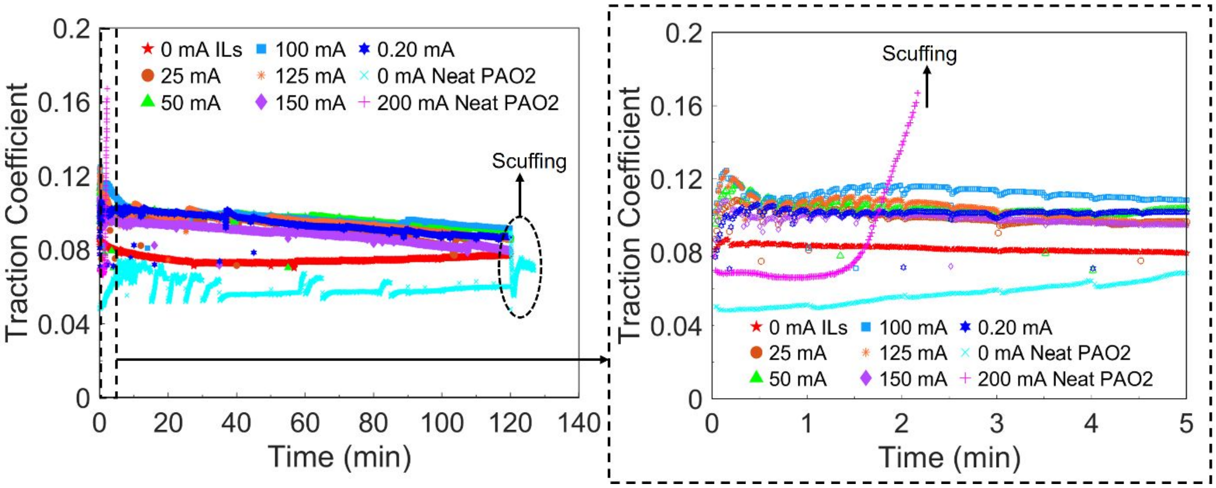

Figure S4. Traction coefficient as a function of time under various current conditions, with

scuffing observed for neat PAO2 under both electrified and non-electrified conditions.

The scuffed surfaces were examined under the optical microscope. As shown in **Figure S5**, wear was evident under both electrified and unelectrified tests. The electrified test specimen displayed pronounced scuffing, consistent with sudden spike in the traction data in **Figure S4**. These observations highlight the importance of additives to prevent scuffing under electrified contact conditions.

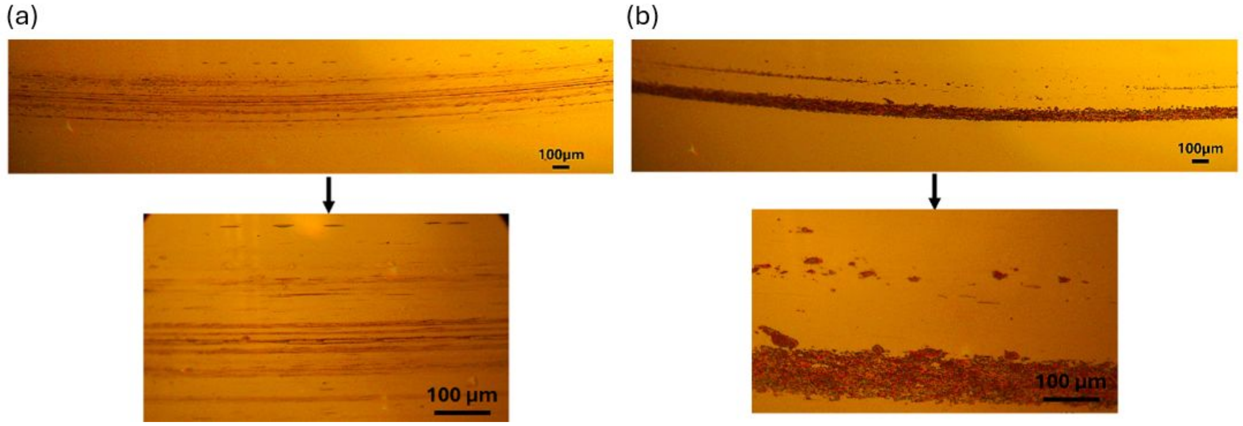

Figure S5. Optical microscopy images of scuffed disc surfaces tested under (a) 0 and (b) 200 mA conditions, with pure PAO2.

S3.3. Topography of tribofilms

Application of electric current led to noticeable changes in the nanoscale topography of the tribofilms. Upon electrification, the running track (tribofilm) became smoother compared to the non-electrified condition (**Figure S6a**). AFM images in **Figure S6b,c** show that the tribofilm/ running track after the 0 A test exhibits deeper wear scars, whereas the 200 mA condition results in a smoother film without significant scarring. This result supports that electric currents promote the deposition of ionic liquid-derived tribofilms, which then reduce wear. This result is consistent with the in-situ tribofilm growth measurements reported in **Figure 2** as well as scanning electron microscopy (SEM) images shown in **Figure 3**.

We also note that the surface roughness depends on the magnitude of the applied current. It progressively increases as the current is raised from 50 to 200 mA, which could be due to pitting of tribofilms, reported in **Figure 3d** and **Section S3.4**.

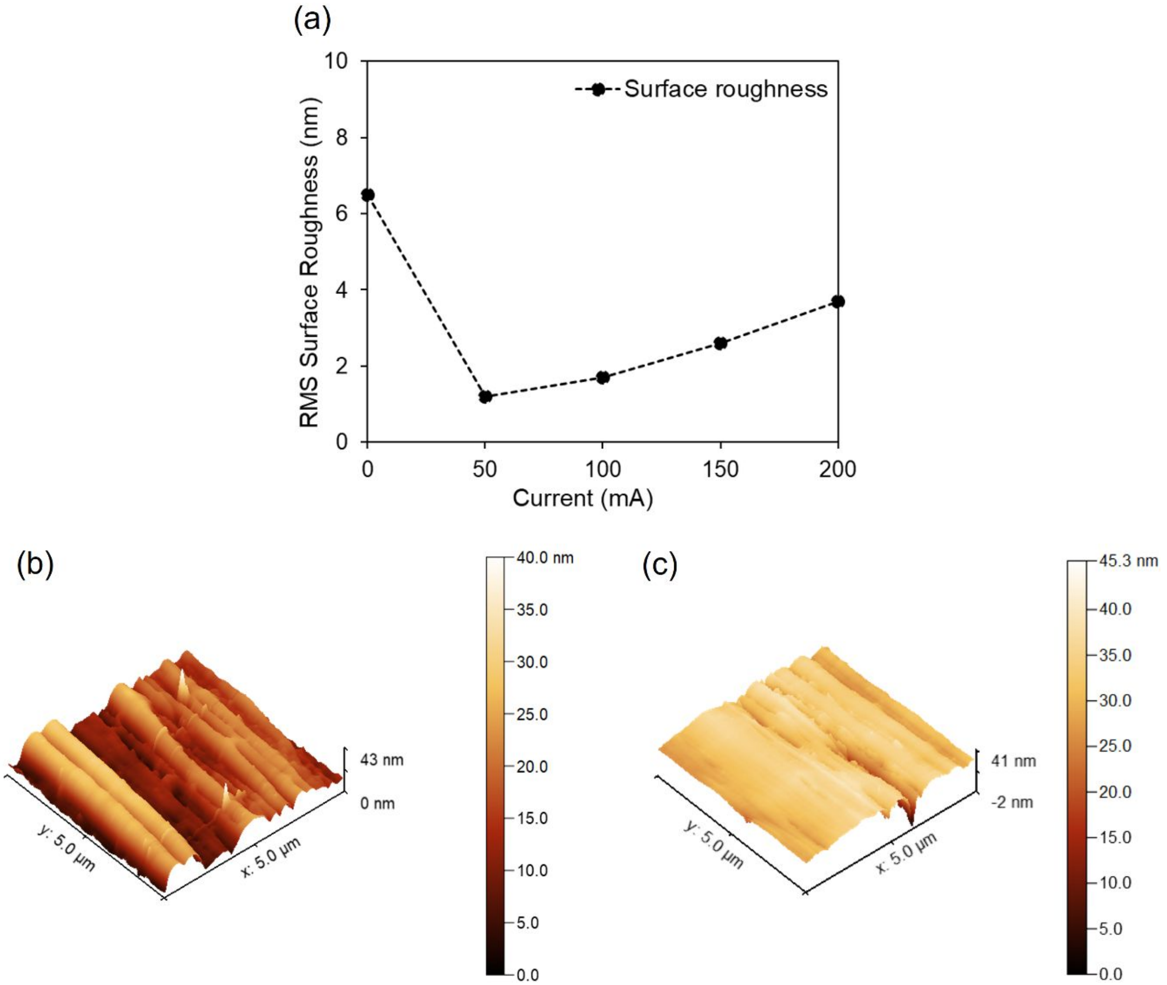

Figure S6. (a) Evolution of surface roughness (RMS) as a function of applied current for tribofilms formed by the ionic liquid additive. AFM scans showing tribofilms formed on disc specimens under (b) non-electrified and (c) 200 mA electrified test conditions.

S3.4. Micropitting at electrified interfaces

SEM analysis was performed to examine the morphology of tribofilms. We observed micron-sized pits in the tribofilms produced under direct currents (**Figure S7**). Based on the SEM images, we calculated the pitting density, i.e., area of pits (obtained using ImageJ) divided by the total area of the micrograph.

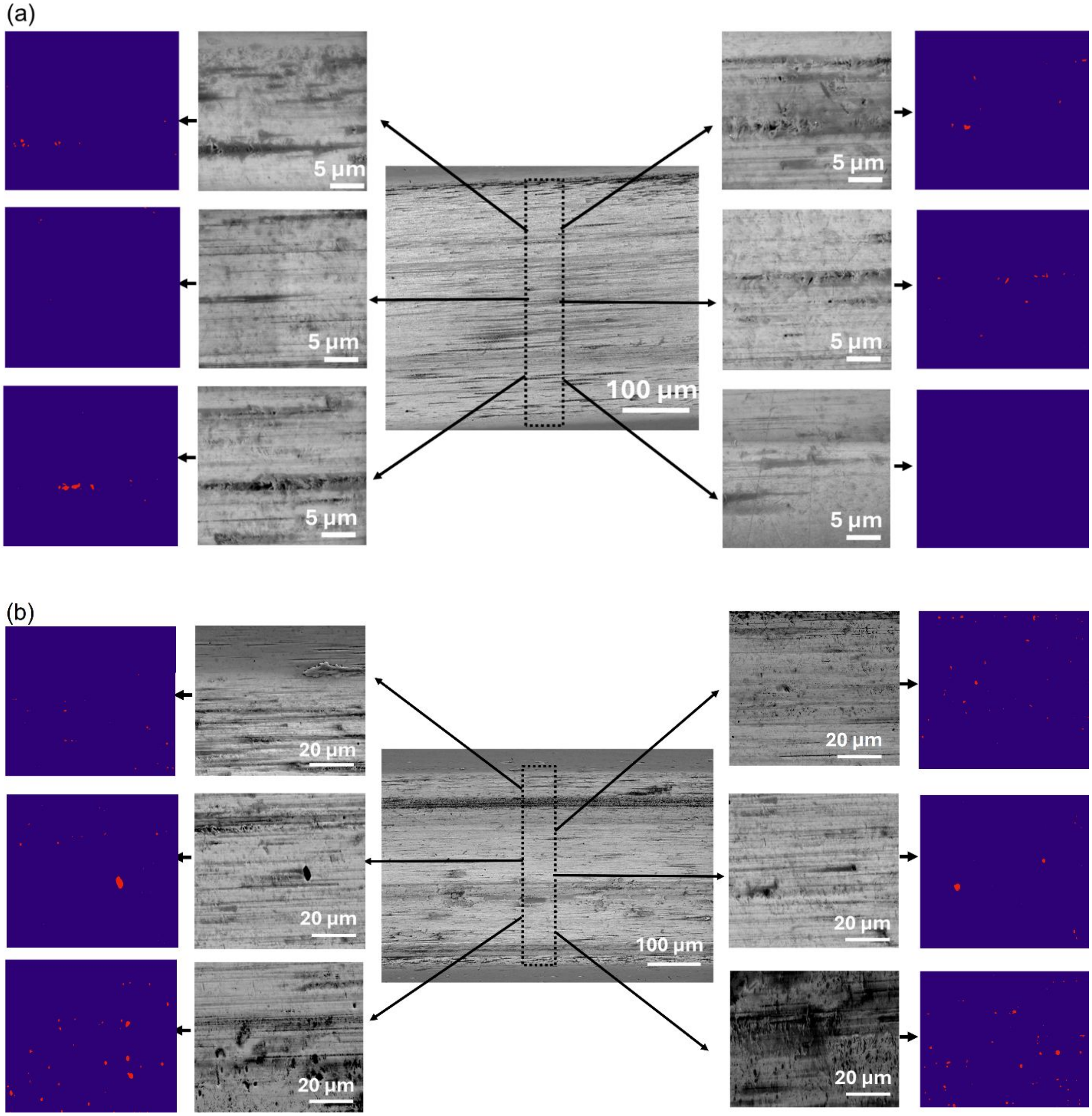
(a)
5 µm
5 µm
5 µm
100 µm
5 µm
5 µm
5 µm
(b)
20 µm
20 µm
20 µm
100 µm
20 µm
20 µm
20 µm

(c)

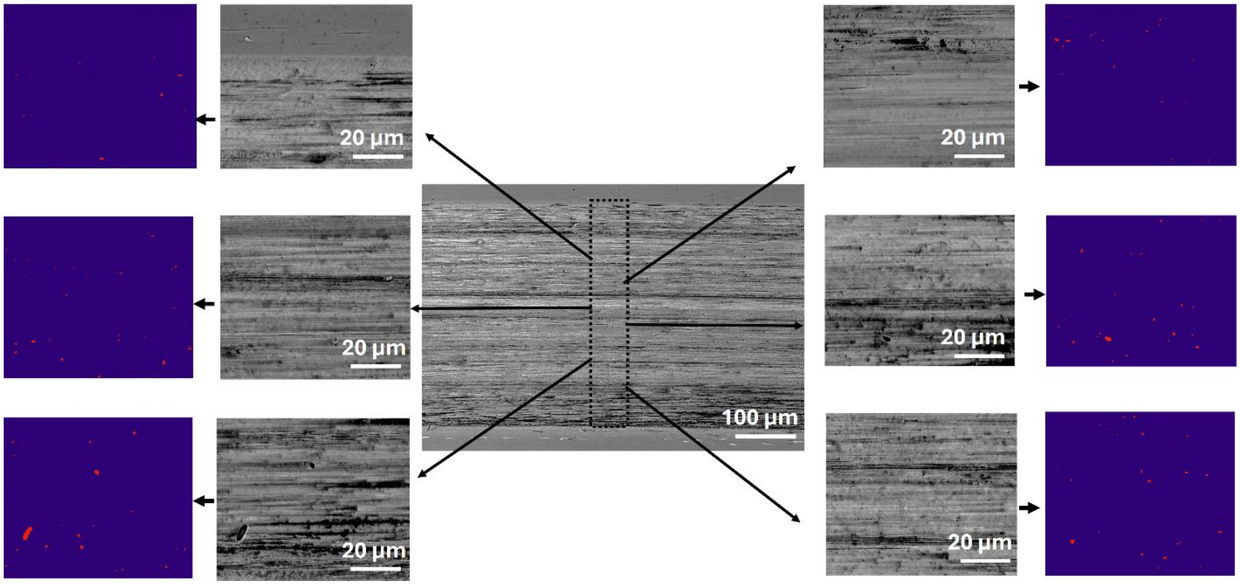
20 µm
20 µm
20 µm
20 µm
20 µm
20 µm
100 µm

(d)

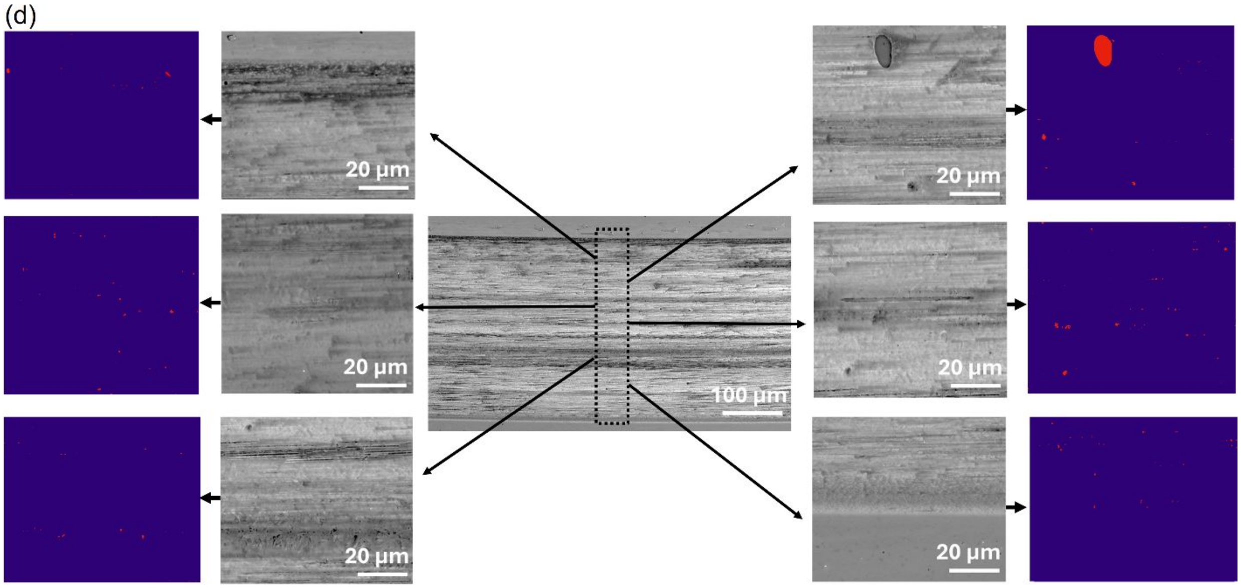
20 µm
20 µm
20 µm
20 µm
20 µm
20 µm
100 µm

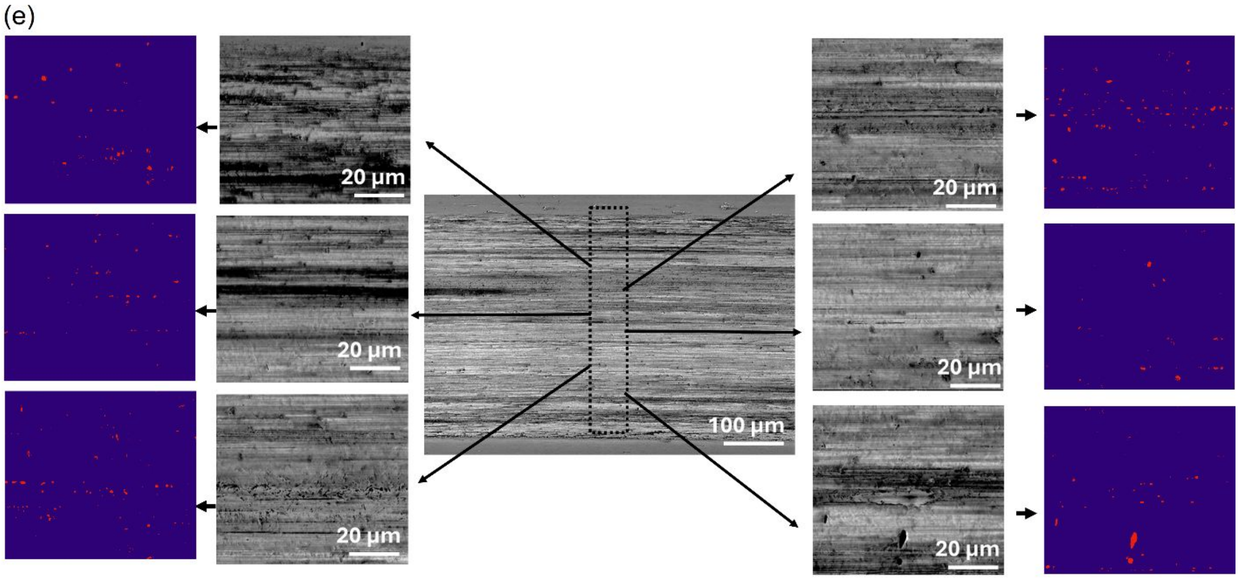

Figure S7. SEM images highlight pitting across the running track (tribofilm) under (a) 25 mA, (b) 50 mA, (c) 100 mA, (d) 150 mA, and (e) 200 mA electrified tests. Note that the scale bar for the 25 mA tribofilm is different than the other tribofilms. The accompanying maps were generated by thresholding SEM micrographs, showing pits as red regions and unpitted area as blue background.

S3.5 XPS P 2p Spectrum of Pure [P_{8888}][DEHP] Ionic Liquid on a Silicon Wafer

To identify the chemical origin of phosphorus-containing species detected in tribofilms, XPS analysis was performed on pure, unreacted [P_{8888}][DEHP] deposited on a silicon wafer (**Figure S8**). The peak at 143 eV is similar to the unidentified peak obtained from the electrified tribofilm in **Figure 4**. Therefore, we conclude that the unidentified peak in Figure 4 of the main paper represents unreacted ionic liquid.

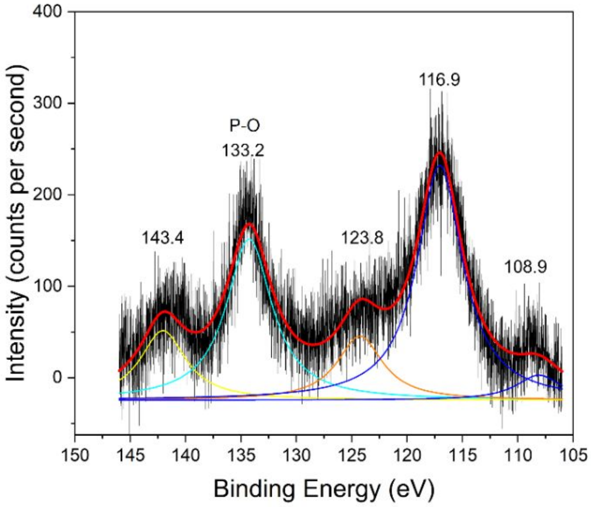

Figure S8. XPS P 2p spectrum of pure [P_{8888}][DEHP] ionic liquid on a silicon wafer.

S4. References

(1) Reddyhoff, T.; Schmidt, A.; Spikes, H. Thermal Conductivity and Flash Temperature. *Tribol Lett* **2019**, *67* (1), 22. https://doi.org/10.1007/s11249-018-1133-8.

(2) Zhu, D.; Wang, J.; Jane Wang, Q. On the Stribeck Curves for Lubricated Counterformal Contacts of Rough Surfaces. *Journal of Tribology* **2015**, *137* (2). https://doi.org/10.1115/1.4028881.

(3) Hamrock, B. J.; Dowson, D. Isothermal Elastohydrodynamic Lubrication of Point Contacts: Part III-Fully Flooded Result. *Journal of Tribology* **1977**, *99* (2), 264–275. https://doi.org/10.1115/1.3453074.

(4) Chapter 7 - Elastohydrodynamic Lubrication. In *Engineering Tribology (Fourth Edition)*; Stachowiak, G. W., Batchelor, A. W., Eds.; Butterworth-Heinemann: Boston, 2014; pp 293–370. https://doi.org/https://doi.org/10.1016/B978-0-12-397047-3.00007-2.

(5) Spikes, H. A. Sixty Years of EHL. *Lubrication Science* **2006**, *18* (4), 265–291. https://doi.org/10.1002/ls.23.

(6) MacLaren, A.; LaMascus, P.; Carpick, R. W. Enhancing the Range and Reliability of the Spacer Layer Imaging Method. *Tribology Letters* **2024**, *72* (3), 95. https://doi.org/10.1007/s11249-024-01890-0.

(7) Brow, R. K.; Tallant, D. R.; Myers, S. T.; Phifer, C. C. The Short-Range Structure of Zinc Polyphosphate Glass. *Journal of Non-Crystalline Solids* **1995**, *191* (1–2), 45–55. https://doi.org/10.1016/0022-3093(95)00289-8.

(8) Luiz, J. F.; Spikes, H. Tribofilm Formation, Friction and Wear-Reducing Properties of Some Phosphorus-Containing Antiwear Additives. *Tribology Letters* **2020**, *68* (3), 75. https://doi.org/10.1007/s11249-020-01315-8.

(9) Gosvami, N. N.; Bares, J. A.; Mangolini, F.; Konicek, A. R.; Yablon, D. G.; Carpick, R. W. Mechanisms of Antiwear Tribofilm Growth Revealed in Situ by Single-Asperity Sliding Contacts. *Science* **2015**, *348* (6230), 102–106. https://doi.org/10.1126/science.1258788.

(10) Ajayi, O. O.; Lorenzo-Martin, C.; Erck, R. A.; Fenske, G. R. Scuffing Mechanism of Near-Surface Material during Lubricated Severe Sliding Contact. *Wear* **2011**, *271* (9–10), 1750–1753. https://doi.org/10.1016/j.wear.2010.12.086.

(11) Ludema, K. C. A Review of Scuffing and Running-in of Lubricated Surfaces, with Asperities and Oxides in Perspective. *Wear* **1984**, *100* (1–3), 315–331. https://doi.org/10.1016/0043-1648(84)90019-X.
